# An integrated review of "unplanned" dialysis initiation: reframing the terminology to "suboptimal" initiation

**DOI:** 10.1186/1471-2369-10-22

**Published:** 2009-08-12

**Authors:** David C Mendelssohn, Christine Malmberg, Bassem Hamandi

**Affiliations:** 1 Humber River Regional Hospital and University of Toronto, Toronto, Canada; 2 Department of Pharmacy, University Health Network, Toronto, Canada; 3 Leslie Dan Faculty of Pharmacy, University of Toronto, Toronto, Canada

## Abstract

**Background:**

Ideally, care prior to the initiation of dialysis should increase the likelihood that patients start electively outside of the hospital setting with a mature arteriovenous fistula (AVF) or peritoneal dialysis (PD) catheter. However, unplanned dialysis continues to occur in patients both known and unknown to nephrology services, and in both late and early referrals. The objective of this article is to review the clinical and socioeconomic outcomes of unplanned dialysis initiation. The secondary objective is to explore the potential cost implications of reducing the rate of unplanned first dialysis in Canada.

**Methods:**

MEDLINE and EMBASE from inception to 2008 were used to identify studies examining the clinical, economic or quality of life (QoL) outcomes in patients with an unplanned versus planned first dialysis. Data were described in a qualitative manner.

**Results:**

Eight European studies (5,805 patients) were reviewed. Duration of hospitalization and mortality was higher for the unplanned versus planned population. Patients undergoing a first unplanned dialysis had significantly worse laboratory parameters and QoL. Rates of unplanned dialysis ranged from 24-49%. The total annual burden to the Canadian healthcare system of unplanned dialysis in 2005 was estimated at $33 million in direct hospital costs alone. Reducing the rate of unplanned dialysis by one-half yielded savings ranging from $13.3 to $16.1 million.

**Conclusion:**

The clinical and socioeconomic impact of unplanned dialysis is significant. To more consistently characterize the unplanned population, the term *suboptimal initiation *is proposed to include dialysis initiation in hospital and/or with a central venous catheter and/or with a patient not starting on their chronic modality of choice. Further research and implementation of initiatives to reduce the rate of *suboptimal initiation *of dialysis in Canada are needed.

## Background

Chronic kidney disease (CKD) is a growing public health concern. At the end of 2005, an estimated 1 in 1,000 Canadians had been diagnosed with end-stage renal disease (ESRD) with almost 61% (19,721 of 32,375) receiving dialysis [1]. Between 1996 and 2005 the incident rate of renal replacement therapy (RRT) rose 36% in Canada from 119 to 162 per million.

Despite advances in nephrological care prior to and after dialysis is initiated, ESRD patients continue to have a high morbidity and mortality, and a significant decline in quality of life. In Canada, the five-year survival of ESRD patients on dialysis ranges from 20% for diabetics over age 65 years to 59% for non-diabetics aged 18-65 years [1]. The mean number of co-morbid conditions in dialysis patients is approximately four per patient, the mean hospital days/patient/year is approximately 15, and self-reported quality of life is far lower than the general population [2].

The cost of treating ESRD in Canada is significant. In 2000, the direct health-care expenditures for ESRD were estimated at $1.3 billion [3]. Patients on dialysis were responsible for approximately two-thirds (69%) of all ESRD expenditures. Although only 0.1% of Canadians had ESRD, these costs represented 1.3% of Canada's total health-care spending [3].

Canadian guidelines recommend referral to a nephrologist for patients with: acute kidney failure; eGFR < 30 mL/min/1.73 m^2^; progressive decline of eGFR; persistent proteinuria; or, inability to achieve treatment targets or other difficulties in the management of CKD [4]. Similarly, the National Kidney Foundation's Kidney Disease Outcome Quality Initiative (NKF/KDOQI) guidelines recommend co-management with a nephrologist at stage 3 CKD (eGFR 30-59 mL/min/1.73 m^2^), and referral at stage 4 (eGFR <30 mL/min/1.73 m^2^) [2].

A recent meta-analysis evaluating timing of referral before starting RRT, has shown that patients referred late to nephrologists have a two-fold higher risk of death compared with early referral (relative risk 1.99; 95% confidence interval (CI), 1.66 to 2.39, p < 0.001) [5]. The duration of hospital stay at the time of initiation of RRT was also greater in the late referred group by an average of 12 days (95% CI, 8.0 to 16.1; p = 0.0007) [5]. A Canadian economic evaluation of early versus late referral of patients with progressive renal insufficiency to a multidisciplinary clinic, showed that early referral is cost-effective and is associated with an incremental cost savings and a reduction in hospital days [6].

Early referral should increase the likelihood that patients initiate dialysis electively, outside of the hospital, with a mature arteriovenous fistula (AVF) or peritoneal dialysis (PD) catheter on the optimal chronic modality of choice. However, unplanned dialysis continues to occur in patients both known and unknown to nephrology services and in both late and early referrals. Recently, Mendelssohn *et al *[7] found that 70% of incident HD patients in Canada start with a central venous catheter (CVC).

The objective of this article is to review the available published literature that examines the clinical and socioeconomic outcomes of unplanned dialysis initiation. The secondary objective is to explore the potential cost implications of reducing the rate of unplanned first dialysis in Canada.

## Methods

The literature review included studies examining the clinical, economic or quality of life outcomes in patients with an unplanned, compared to those with a planned, first dialysis. Unplanned dialysis was broadly defined as any patient who received unanticipated dialysis regardless of location or previous referral status to nephrologists. Terms considered synonymous to unplanned dialysis were unscheduled, unprogrammed, urgent, and emergent. The search strategy included MESH headings "kidney failure, chronic" or "dialysis." These headings were combined with the non-MESH headings "planned" or "unplanned" or "emergent" or "unscheduled" or "non-programmed" and the MESH heading "time factors". Databases used were MEDLINE and EMBASE from inception to 2008. The reference lists of published papers examining the impact of early referral status were examined for additional relevant studies. Reviews, editorials, practice guidelines, and studies conducted in children were not included. Data from each study were described in a qualitative manner. Crude cost impact estimates of unplanned dialysis in Canada were performed based on data from the Canadian Organ Replacement Registry Report (CORR) and the Canadian Institute for Health Information (CIHI).

## Results

### Description of Studies

Described in Table 1, the eight studies included in this review were prospective observational, or retrospective, enrolling 109 to 2815 patients, with follow-up periods ranging from 8 weeks to three years. Outcomes included clinical status (e.g. laboratory parameters) at baseline [8,9], mortality [10-13], hospitalization [10,12] and quality of life [14,15]. Three studies assessed only baseline status at first dialysis [8,9,15]. One study reported economic outcomes [12].

**Table 1:** Studies summarizing the outcomes of planned and unplanned dialysis.

Author, year	Country, Design, N, Follow-up	Terminology & Definition of Unplanned Dialysis	Proportion unplanned dialysis start (%)	Main Outcome Measure
Buck et al, 2007	UK Retrospective N = 109 Follow-up: none - survey at dialysis start	Known acute - known to renal services for >4 months and used hemodialysis catheter or required emergency admission to start dialysis.	45%	Albumin, hemoglobin, serum creatinine, urea and phosphate.

Caskey, 2003	7 European countries, Prospective N = 196 Follow-up: 8 weeks	Unplanned HD or PD - referred to nephrologists at least 1 month and first dialysis not planned in advance (patients without a creatinine > 300 mmol/L upon referral were excluded)	36%	QOL measured by Visual analogue scale and SF-36

Castellano, 2006	Spain Retrospective N = 117 Follow-up: 6 months	Non-programmed - started for an emergency condition or not appropriate to delay for more than 24 hours	44%	Hospital admission. Death.

Couchoud, 2007	France Retrospective N = 2816 Follow-up: 2 years	Unplanned hemodialysis - begun in an emergency basis (life threatening requiring dialysis within 24 hours) in patients over 75 years	39%	Death.

Gorriz, 2002	Spain Retrospective N = 362 Follow-up: 3 years	Unplanned dialysis - any dialysis start without a vascular or peritoneal access ready to use	49%	Hospitalization Death Direct medical cost (dialysis, hospitalization, physician) during first 6 months.

Loos, 2003	France Cross-sectional observational N = 169 Follow-up: none, survey at baseline.	Unplanned dialysis in patients over 70 years - not clearly defined	46%	QOL SF-36

Marron, 2005	Spain Retrospective N = 1504 Follow-up: none, survey at dialysis start	Non-planned - not scheduled, even if a permanent dialysis access in place.	46%	Age, pre-dialysis follow-up time, rate of PD, rate of permanent access, renal function, biochemical status.

Metcalfe, 2000	Scotland Prospective N = 532 Follow-up: 90 days	Unplanned - follow up by nephrologists for 1 month, steady progression to end stage, no permanent access	24%	Mortality during first 90 days.

### Definition of Unplanned Dialysis

The majority of studies used the term "unplanned". Two studies used the terms "known acute" and "non-programmed" [8,14]. With the exception of three studies that included only patients referred to nephrology prior to first dialysis [8,13,14], most enrolled patients regardless of referral status to nephrology. Three studies defined unplanned dialysis as starting in a life threatening situation [8,10,11]. Two other studies defined unplanned as those patients that did not have a permanent access device in place [12,13].

### Baseline Clinical Status

Two studies were designed to examine the baseline characteristics of patients starting unplanned dialysis. The first study was a retrospective survey conducted at a large regional renal network [8]. *Elective *patients commenced RRT in the outpatient setting using either an AVF, PD catheter or by pre-emptive transplantation. *Known Acute *patients were known to renal services for more than four months and started dialysis with a hemodialysis catheter or during an emergency hospital admission. Patients had been known for a median time of three years with no statistical difference in the length of time between the two groups. At the start of RRT, *known acute *patients had statistically significant lower concentrations of serum albumin and hemoglobin than *elective *patients. This group also had higher serum creatinine, urea and phosphate concentrations compared with the *elective *group.

A multicentre retrospective review examined the factors affecting a *planned *versus *non-planned *start of dialysis [9]. A *planned *start was a scheduled outpatient initiation of dialysis with the use of a permanent vascular or peritoneal access. Conversely, a *non-planned *start was unscheduled and included patients with and without a permanent dialysis access in place. Forty-six percent started dialysis in a *non-planned *fashion, although half of these patients had been followed by a nephrologist for at least 3 months. *Planned *starts were associated with a younger age, longer renal and pre-dialysis follow-up, more patient education, more medical visits, more follow-up by specific ESRD units, more permanent access (including PD), and better renal function and biochemical status at the start of dialysis.

Three other studies evaluated baseline clinical status and outcomes over a longer period of time. Similar to previous findings [8,9], planned dialysis was associated with higher baseline levels of serum hemoglobin, calcium and albumin and lower baseline levels of serum urea, creatinine and phosphate [10,12,13]. Gorriz *et al *(2002) also showed that unplanned dialysis initiation was associated with uremic symptoms, fluid overload and increased transfusion requirements [12]. Unplanned patients were older, and had a shorter follow-up period of 3 months [13]. In both studies, unplanned patients had a higher co-morbidity index [12,13].

### Morbidity and Mortality

Four studies examined morbidity and mortality outcomes over study periods ranging from 6 months to three years. Duration of hospitalization was consistently longer for unplanned dialysis. Mortality was also higher in the unplanned dialysis group in all [11-13] but one study [10].

A retrospective Spanish study classified 117 patients as either *programmed *or *non-programmed *[10]. A patient was *programmed *when dialysis was planned with time, and *non-programmed *when the first dialysis was started for an emergency condition or was not appropriate to delay for >24 hours. *Non-programmed *dialysis occurred among 52/117 patients. *Non-programmed *patients were more likely to be admitted to hospital for initiation of dialysis (90.4% vs. 6.1%; p < 0.001) and during the first 6 months (48% vs. 15.3%; p < 0.001). The duration of hospitalization was longer for the *non-programmed *group (23.6 vs. 3.0 days; p < 0.001). The 6-month mortality rate was not significantly different although a trend towards higher mortality was observed (11.5% vs. 4.6%) in the *non-programmed *versus *programmed *groups, respectively.

A retrospective analysis from the French Renal Epidemiology and Information Network (REIN) registry [11], studied the clinical and laboratory factors associated with choice of first treatment and two-year survival in patients older than 75-years. *Unplanned HD *was defined as any first HD begun on an emergency basis in life threatening circumstances requiring dialysis within 24 hours. A total of 1110/2816 HD patients started dialysis in an *unplanned *manner. *Unplanned HD *was associated with a significantly higher 2-year mortality rate than *planned *HD (39.1% vs. 25.8%, adjusted HR 1.5).

Gorriz *et al *[12] classified patients initiating RRT according to whether a vascular or peritoneal access was ready to use (*planned dialysis*), or not (*unplanned dialysis*). Of the 362 Spanish patients studied, 176 (48.6%) were considered *unplanned*. The *unplanned *group was associated with an increased rate of hospitalization secondary to the need for emergent dialysis (90.3% vs. 16.7%; p < 0001), requiring a significantly longer duration of initial admission (17.7 vs. 4.0 days; p < 0.001). *Unplanned dialysis *was also associated with increased 6-month mortality (10.2% vs. 3.2%; p = 0.015), and three-year mortality (36.9% vs. 24.2%; p = 0.006).

Metcalfe *et al *[13] performed a prospective nationwide study of all patients commencing RRT in Scotland over a one-year period. Patients were classified as *planned *when a permanent vascular or peritoneal access was ready for use at the first RRT, and *unplanned *when follow-up by a nephrologist occurred for <1 month, with steady progression to ESRD, and no permanent access. An *unplanned *presentation occurred in 129/532 patients, and relative to those with a *planned *presentation, these patients were more likely to die within 90 days (12.4% vs. 3.1%; p = 0.001), with a longer hospital admission (median, 9 vs. 3 days; p < 0.001) at the time of RRT initiation.

### Economic Outcomes

The Gorriz *et al *study described above evaluated the direct medical costs (dialysis, hospital, and physician costs) of unplanned dialysis initiation [12]. On a per patient basis, the direct costs associated with an unplanned initiation were 4.4 times that of a planned initiation during the first six months of dialysis. This difference was due mainly to a higher number of dialysis sessions during hospitalization and a higher incidence of hospitalization.

### Quality of Life Outcomes

Caskey *et al *[14] examined QoL after 8 weeks among a cohort of 196 European HD and PD patients referred to a nephrologist >1 month before their first dialysis. *Planned *dialysis patients had a previously documented serum creatinine >300 mmol/L and a first dialysis arranged in advance and not performed urgently for life-threatening renal insufficiency. Seventy of the 196 (36%) patients had an *unplanned *first dialysis. The Visual Analogue Scale (VAS) was significantly higher for *planned *versus *unplanned *patients (60.7 vs. 54.2; p = 0.03). *Planned *patients also had a higher Short Form Health Care Survey 36 (SF-36) mental component summary score (45.4 vs. 39.7; p = 0.003), role emotional scores (58.0 vs. 30.9; p = 0.003) and mental health scores (63.7 vs. 54.6; p = 0.01) as compared to *unplanned *patients. Multiple linear regression showed that *planned *first dialysis had an independent increase on QoL (VAS; SF-36's mental summary score, physical functioning, role physical, general health, role emotional and mental health).

A cross-sectional, observational study of 169 elderly French ESRD patients, assessed the QoL impact of *planned *versus *unplanned *first dialysis using the SF-36 questionnaire at start of dialysis [15]. *Unplanned *dialysis occurred among 46% of the patients. Pulmonary and peripheral edema, digestive disorders, and anorexia were significantly more common in *unplanned *versus *planned *first dialysis. Sodium and hematocrit levels were also significantly lower in the *unplanned *dialysis group. Adjusting for other factors, the QoL scores were significantly lower for the physical function (24.9 vs. 37.4; p = 0.01) and vitality (26.3 vs. 34.2; p = 0.01) dimensions among *unplanned *versus *planned *patients, respectively.

### Economic Impact Estimates

The Canadian Institute for Health Information (CIHI) has reported direct hospital costs of $16,740 for unplanned admission versus $3,485 for a planned admission related to dialysis [16]. The 2005 incident dialysis rate in Canada was 162/1,000,000 or approximately 5,285 new dialysis patients (based on a population of 32,623,490) [1]. Assuming a conservative incident unplanned dialysis rate of 30% (a range of 24-49% was reported in this literature review), a 100% rate of initial hospitalization for all unplanned dialysis starts, and a 50% rate of re-hospitalization within one year [10], the total annual burden to the Canadian healthcare system of unplanned dialysis in 2005 would be approximately $33 million in direct hospital costs alone (Table 2).

**Table 2:** Estimated annual burden in hospital costs of unplanned dialysis in 2005

Incident dialysis cases in Canada (2005)^a^	5,285
Rate of unplanned dialysis	30%

Number of unplanned dialysis starts (5,285 × 0.3)	1586

Annual rate of readmission in unplanned dialysis population	50%

Annual number of readmissions in unplanned dialysis population (1,585 × 0.5)	793

Cost of an unplanned hospitalization for dialysis^b^	$16,740

Cost of other hospitalization with urinary system diagnosis^b^	$8,841

Cost of hospitalizations secondary to unplanned dialysis starts (1,585 × $16,740)	$26,541,270

Cost of re-hospitalizations in unplanned dialysis population (793 × $8,841)	$7,008,703

Total annualized hospital costs related to unplanned dialysis	$33,549,973

^a^Canadian Institute for Health Information, Canadian Organ Replacement Registry Report Volume I: Dialysis and Renal Replacement (Ottawa: CIHI, 2007).

^b^Canadian Institute for Health Information, The Cost of Hospital Stays: Why Costs Vary (Ottawa: CIHI, 2008).

As shown in Table 3, by conservatively assuming that planned first dialysis requires a planned hospital admission ($3,485) and a significantly lower rate of re-hospitalization within one year (10%), the annual hospital costs avoided by preventing one unplanned dialysis start is an estimated $16,791. Reducing the rate of unplanned dialysis by one-half (30% to 15%) or by two-thirds (30% to 10%), yields savings of $13.3 and $17.7 million, respectively (Table 4). Reframing the analysis without an initial planned hospitalization, shows that the annual hospital costs avoided by preventing one unplanned start is $20,276 (Table 3) with even greater savings of $16.1 and $21.4 million when that rate is reduced by one-half and two-thirds, respectively (Table 4).

**Table 3:** Estimated annual hospital cost avoidance by preventing one unplanned dialysis start

	Hospitalization required for first planned dialysis	Hospitalization NOT required for first planned dialysis
Cost of an unplanned hospitalization for dialysis^a^	$16,740	$16,740

Annual rate of readmission in unplanned dialysis population	50%	50%

Cost of other hospitalization with urinary system diagnosis^a^	$8,841	$8,841

Annual hospital cost of unplanned dialysis ($16,740 + (0.5*$8,841))	**$21,161**	**$21,161**

Cost of a planned hospitalization for dialysis^a^	$3,485	$0

Annual rate of readmission in planned dialysis population	10%	10%

Annual hospital cost of planned dialysis (cost of planned hospitalization + (0.1*$8,841))	**$4,369**	**$884**

**Annual hospital cost avoidance by preventing one unplanned dialysis start**	**$16,791**	**$20,276**

^a^Canadian Institute for Health Information, Canadian Organ Replacement Registry Report Volume I: Dialysis and Renal Replacement (Ottawa: CIHI, 2007).

**Table 4:** Projected total annual hospital cost avoidance by reducing the rate of unplanned dialysis in Canada

Reducing the rate of unplanned dialysis from 30% to:	Hospitalization required for first planned dialysis	Hospitalization NOT required for first planned dialysis
20%	$8,874,255	$10,716,077

15%	$13,311,382	$16,074,116

10%	$17,748,510	$21,432,155

## Discussion

This review of eight studies comparing clinical, economic and patient reported outcomes in 5,805 European dialysis patients has shown that duration of hospitalization and mortality is higher for the unplanned versus planned population. Patients undergoing a first unplanned dialysis have significantly worse laboratory parameters at baseline compared with patients undergoing planned dialysis. As well, QoL was significantly worse in unplanned dialysis patients both at baseline, and as early as eight weeks after starting dialysis. The studies reviewed in this article reported rates of unplanned dialysis ranging from 24-49%.

Some patients may have primary care physicians that underestimate the potential benefits of dialysis and/or the length of time required to optimally prepare a patient for dialysis [8]. Educational efforts targeting primary care givers, explaining the clinical, economic, and quality of life benefits of the timely dialysis planning are needed to ensure that all patients with renal failure that potentially require dialysis are referred to a nephrologist in a timely manner. The Canadian Society of Nephrology has a policy document and implementation strategy intended to achieve timely referral of appropriate patients [4].

Pre-dialysis education seems to be significant in determining whether patients have an optimal or suboptimal dialysis start [9]. Patients attending multidisciplinary pre-dialysis clinics were more likely to present with a functioning permanent vascular access at dialysis initiation (48% vs. 5%; p < 0.01) [17]. Moreover, they had fewer hospitalizations at 1-year (7.0 vs. 69.7 d/patient/y; p < 0.01), with fewer deaths at 1-year (2% vs. 23%; p < 0.01) [17]. In a Cox-adjusted linear regression model, non-clinic pre-dialysis care was shown to be an independent predictor of death during therapy (RR 2.9; p = 0.011) [17]. A matched-cohort study evaluating the effectiveness of multidisciplinary care (MDC), showed a 50% mortality risk reduction for MDC compared with non-MDC (HR 0.50; 95% CI, 0.35 to 0.71) [18]. A trend towards a reduction in risk for all-cause and cardiovascular-specific hospitalizations was observed (p = NS). The opportunity to educate patients/caregivers leading to informed decisions may improve QoL and decrease economic resource utilization since patients referred early may be more likely to choose peritoneal rather than hemodialysis [19].

Whether to advise a patient to undergo an attempt to place an AVF is a complex decision that requires a nephrologist's anxious consideration. On the one hand, it is documented in the literature that there is a subset of patients who die with a functional AVF or PD catheter who never started dialysis [20]. On the other hand, there is a compelling moral and ethical obligation to avoid long term catheter based vascular access [21]. Selecting only patients with progressive nephropathy who will need to initiate HD is an imprecise science at present and requires considerable clinical judgement.

One challenge with comparing outcomes across the studies reviewed in this article is the variability in the definition of unplanned dialysis. Unplanned dialysis occurs in patients both known and unknown to nephrology services and in both late and early referrals. Some researchers defined unplanned dialysis only if it was started in a life threatening situation [8,10,11]. Other researchers also included an element of timing of nephrology referral (e.g. 1 to 4 months) [8,13,14] or the lack of a ready to use vascular or peritoneal access [8,9,12] in the definition of unplanned dialysis. We suspect that our search has missed articles because the terms planned and unplanned dialysis starts are difficult to define and inconsistently used in the literature.

To improve evaluation of the effectiveness of strategies for reducing the incidence of unplanned dialysis, a more consistent definition is required. We propose the term *suboptimal initiation *to include all patients starting in hospital and/or with a central venous catheter, and/or not starting on their chronic modality of choice. In contrast, an *optimal start *occurs when patients initiate dialysis electively in an outpatient setting with a mature AVF or PD catheter, on the patient's chosen chronic dialysis modality. We believe this definition is simple and precise, will be accepted by clinicians and researchers, and can be more consistently applied.

Sub-optimal dialysis initiation is surprisingly common. In Canada, 70% of incident patients start with a CVC [7]. In addition, 55% of patients attending a multidisciplinary pre-dialysis clinic at a Toronto hospital did not have a functioning permanent vascular access at the time of starting hemodialysis [17]. Another Canadian cohort study conducted in 15 dialysis centres across 7 provinces during 1998-9, revealed that only 66% of those known to nephrologists had a permanent access in place [22]. Based on these studies, the rate of sub-optimal initiation in Canada is potentially between 55-70%. The estimated cost impact of suboptimal dialysis initiation in this analysis (which is based on a literature rate of 30%) is likely a marked underestimation.

Once referred to a nephrologist, Canadian clinical practice guidelines recommend monitoring renal function and nutritional status every three months, although the actual frequency of clinical evaluation should still be based on clinical judgment [4]. This monitoring frequency is thought to be sufficient to detect patients with a more rapid rate of decline in renal function. It may also permit specific and targeted interventions to slow the decline in renal function or, alternatively, speed preparation for dialysis. However, even in the studies including only patients already referred to a nephrology service, suboptimal dialysis initiation was associated with worse laboratory parameters at baseline and an increase in hospitalization and mortality, suggesting that early dialysis referral does not guarantee optimal care [4,13,14,23].

It is disturbing that suboptimal initiation occurs commonly even when patients are referred to a nephrologist early. A preliminary list of causes of suboptimal dialysis initiation despite early referral includes a) acute on chronic kidney disease, b) patient induced delays and indecision, c) barriers to surgical resources, d) suboptimal nephrology care and e) lack of dialysis resources to accommodate new patients. We recommend that the factors on this list (and possibly others) need to be investigated and quantified so that new approaches can be developed to overcome them.

## Conclusion

This review of eight studies comparing outcomes in 5,805 European dialysis patients has shown that duration of hospitalization and mortality is higher patients undergoing suboptimal initiation. These patients have significantly worse laboratory parameters at baseline, and lower QoL compared with patients initiating dialysis in an optimal fashion.

In Canada, costs associated with suboptimal dialysis initiation are significant. By reducing the estimated rate of unplanned dialysis by one-half, a projected $13.1 to 16.1 million in hospital costs could be avoided. This estimate is conservative since it did not include direct medical costs outside of the hospital and indirect costs such as loss of quality of life and productivity. Further research and initiatives to reduce the rate of suboptimal dialysis initiation in Canada are needed.

## Competing interests

DM has served on advisory boards and has received honorariums for lecturing from Amgen, Baxter, Genzyme, Ortho Biotech, Roche, Shire and several BP medication companies. CM and BH have acted as consultants for Ortho Biotech Canada. The results of this paper have not been presented or published elsewhere, in whole or in part.

## Authors' contributions

CM and BH performed the integrative review and wrote the first draft. DM reviewed the analysis, and wrote the final draft. All authors contributed to the conception and design, and read and approved the final manuscript.

## Pre-publication history

The pre-publication history for this paper can be accessed here:

http://www.biomedcentral.com/1471-2369/10/22/prepub
